# Draft Genome Sequence of Multidrug-Resistant *Enterococcus faecium* Clinical Isolate VRE3, with a Sequence Type 16 Pattern and Novel Structural Arrangement of Tn*1546*

**DOI:** 10.1128/genomeA.00871-15

**Published:** 2015-08-13

**Authors:** Saeed Khan, Kidon Sung, Bernard Marasa, Seonggi Min, Ohgew Kweon, Mohamed Nawaz, Carl Cerniglia

**Affiliations:** a Division of Microbiology, National Center for Toxicological Research (NCTR), U.S. Food and Drug Administration, Jefferson, Arkansas, USA; b Division of Microbiological Assessment, Center for Drug Evaluation and Research (CDER), U.S. Food and Drug Administration, Silver Spring, Maryland, USA; c Office of Scientific Coordination, NCTR, U.S. Food and Drug Administration, Jefferson, Arkansas, USA

## Abstract

Multidrug-resistant *Enterococcus faecium* has emerged as a nosocomial pathogen that may infect the body at various sites, including the gastrointestinal tract, and has serious implications in human health and disease. Here, we present the draft genome sequence of clinical strain VRE3, which exhibited a sequence type 16 (ST16) pattern and carried truncated Tn*1546*, a mobile genetic element encoding a high level of vancomycin resistance.

## GENOME ANNOUNCEMENT

*Enterococcus faecium* is a Gram-positive nonmotile bacterium that constitutes part of the normal microbiota in both animal and human gastrointestinal tracts (1) and causes severe to life-threatening infections (2, 3). *E. faecium* VRE3 is a vancomycin-resistant clinical isolate obtained from Brooks Air Force Base, San Antonio, TX, USA. It lacks the v*anR*, *vanS*, *orf1*, and *orf2* elements of Tn*1546* (4). The *vanR* and *vanS* were replaced by *orf29*, *orf30*, and *orf31* of pRUM-like plasmid (4) and were observed in contig 31. More recently, VRE isolates have been found to possess an altered left end of Tn*1546*, partially deleted *orf1*, and insertions between the *vanS-vanH* and the *orf2*-*vanS* genes (5, 6). A comparative data analysis indicated that VRE3 has novel features in Tn*1546* that make it an interesting candidate strain to study the alternative regulatory mechanism for vancomycin resistance.

The genomic DNA of *E. faecium* VRE3 was extracted by using a Master Pure Gram-positive DNA purification kit (Epicentre Biotechnologies). TruSeq DNA library preparation and paired-end cluster kits (Illumina) were used for the DNA preparation and cluster generation. The Illumina HiSeq2500 system was used for sequencing. *De novo* assembly of a total of 25,585,258 high-quality paired-end reads, 100 bp in length, was conducted using the CLC Genomics Workbench version 6.5.1 (CLC Bio), and further genome annotation was performed using the GeneMarkS+ method in the NCBI Prokaryotic Genome Annotation Pipeline (http://www.ncbi.nlm.nih.gov/genome/annotation_prok). The draft genome sequence of *E. faecium* VRE3 was 2,820,231 bp in length with a G+C content of 37.8%, which is distributed in 142 contigs (*N*_50_ length, 60,432; average coverage, 40.0×) with 2,689 coding sequences (CDS) and 52 RNAs.

### Nucleotide sequence accession numbers.

This whole-genome shotgun project has been deposited at DDBJ/EMBL/GenBank under the accession number JSET00000000. The version described in this paper is the first version, JSET01000000.
